# Reactive Infectious Mucocutaneous Eruption (RIME) in an Adult Male With Mycoplasma pneumoniae: A Case Report

**DOI:** 10.7759/cureus.78301

**Published:** 2025-01-31

**Authors:** Saud Alawad, Nawaf Alsaeed, Bailey Burnette, Mark Colantonio, Lindsay Kasson

**Affiliations:** 1 Internal Medicine, West Virginia University School of Medicine, Morgantown, USA

**Keywords:** conjunctivitis, corticosteroids, dysuria, extrapulmonary manifestations, mucositis, mycoplasma pneumoniae, rime, targetoid rash

## Abstract

*Mycoplasma pneumoniae* (*M*. *pneumoniae*) typically presents with respiratory symptoms and is considered a common cause of community-acquired pneumonia. *M*. *pneumoniae* infection occasionally presents with extrapulmonary manifestations, among which reactive infectious mucocutaneous eruption (RIME) represents an infrequent but clinically significant complication. This dermatological sequela, while uncommon, underscores the systemic potential of *M. pneumoniae* infections beyond their typical respiratory presentation. Herein, a 24-year-old man presented to the hospital with a recent diagnosis of mycoplasma pneumonia and symptoms that included severe mucositis, conjunctivitis, dysuria, and targetoid skin lesions. The initial, broad therapy with antibiotics, including doxycycline and azithromycin, was quickly followed by exacerbation of his symptoms, requiring systemic corticosteroid therapy. There was a significant clinical improvement with the intravenous use of methylprednisolone within three days. This case represents an extremely rare age group, echoing that early recognition and management of RIME are of utmost importance.

## Introduction

*Mycoplasma pneumoniae* is an atypical bacterium. It is the primary cause of respiratory infections, especially among children and young adults, often giving rise to community-acquired pneumonia (CAP). *M. pneumoniae* infections are responsible for 10-30% of CAP, particularly in the younger population, with patients commonly presenting with fever, malaise, and a persistent cough [1]. Although respiratory symptoms are common, *M. pneumoniae* infection is associated with numerous extrapulmonary complications in as many as 40% of infected cases [2]. Extrapulmonary manifestations, including the central nervous and cardiovascular systems, may range from mild to severe, and skin and mucous membrane involvement rarely [2].

Reactive infectious mucocutaneous eruption (RIME) is exceedingly uncommon in adults; however, it is a significant complication of *M. pneumoniae* infection, consisting of a triad of skin eruptions, including mucositis of the oral, ocular, and genital mucosa and targetoid or erythematous skin eruptions. It was previously called mycoplasma-induced rash and mucositis (MIRM). The definition has changed to distinguish it from Stevens-Johnson Syndrome (SJS) and erythema multiforme (EM) because these diseases also present similar kinds of mucocutaneous manifestations [3]. The central distinguishing feature of RIME is its infectious etiology. Epidemiological data indicate higher incidence rates of *M. pneumoniae* infections in Europe and Asia compared to America and Oceania [4]. While *M. pneumoniae* remains a prominent etiological agent, other pathogens such as *Chlamydophila pneumoniae, *human metapneumovirus*, *and rhinoviruses have been implicated in the pathogenesis of RIME. In contrast, SJS and EM are predominantly associated with adverse drug reactions [3].

While RIME is reported mainly in pediatric populations, it may well occur in adults, though far less common. Its rarity and overlapping clinical features with other dermatologic entities make diagnosing RIME in adults a real challenge. The supposed pathogenesis involves an immune-mediated reaction against *Mycoplasma* antigens, leading to generalized inflammation of the mucosa and skin [5]. The case described herein concerns a 24-year-old male who developed RIME after a confirmed infection with *M. pneumoniae*. The disease did not resolve by itself following the use of antibiotics but instead required systemic corticosteroid therapy.

## Case presentation

A 24-year-old male school bus driver with no significant past medical history and no known allergies was admitted to the emergency department due to complaints of productive cough, persistent headache, and malaise, that had been ongoing for seven days. He denied any recent travel or exposure to others with similar symptoms. The patient was febrile to 38.1 C (100.5 F), had a blood pressure of 120/72, a respiratory rate at 20 breaths per minute, and a heart rate of 92 beats per minute with an oxygen saturation of 96% on room air. He had clear breath sounds bilaterally to lung auscultation without wheeze or crackles.

**Figure 1:**
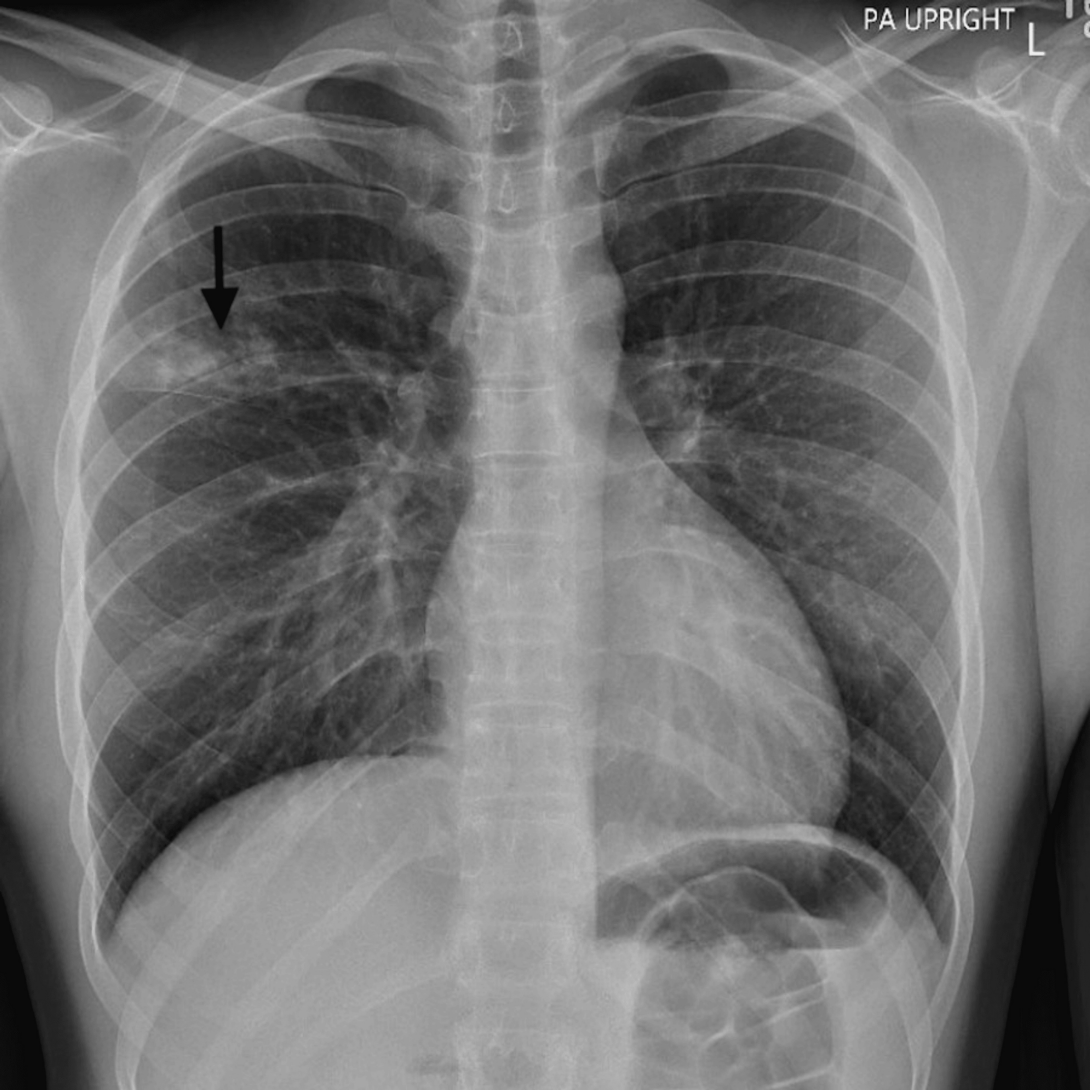
Chest X-ray at presentation showing opacity in the right upper lobe consistent with pneumonia

Initial laboratory tests showed a WBC count of 14.3 x 10^9/L, with 70% neutrophils and 6.3% eosinophils, and platelets level at 818 x 10^9/L, which is concerning for the inflammatory process in the current presentation (Table 1). The basic metabolic panel was unremarkable. The respiratory viral panel was positive for *M. pneumoniae*, as the chest X-ray showed opacity in the right upper lobe, which was consistent with pneumonia (Figure 1). He was diagnosed with *M. pneumoniae* and discharged on oral doxycycline 100 mg twice daily for seven days, with instructions to return if symptoms failed to improve.

**Table 1:** Laboratory tests WBC: white blood cells; RBC: red blood cells; HGB: hemoglobin; HCT: hematocrit; MCV: mean corpuscular volume; MCH: mean corpuscular hemoglobin; MCHC: mean corpuscular hemoglobin concentration; RDW-CV: red cell distribution width-coefficient of variation; MPV: mean platelet volume; PMNs: polymorphonuclear leukocytes

Test	Initial results	Results after 5 days	Reference range and units
WBC	14.2	12.8	3.7 - 11.0 x10^3/uL
RBC	4.46	4.45	4.50 - 6.10 x10^6/uL
HGB	14.3	14.1	13.4 - 17.5 g/dL
HCT	40.8	39.3	38.9 - 52.0 %
MCV	91.5	88.3	78.0 - 100.0 fL
MCH	32.1	31.7	26.0 - 32.0 pg
MCHC	35	35.9	31.0 - 35.5 g/dL
RDW-CV	12	11.9	11.5 - 15.5 %
Platelet count	818	916	150 - 400 x10^3/uL
MPV	9	8.8	8.7 - 12.5 fL
PMNs	70.2	86.6	%
Lymphocytes	14.1	8.9	%
Monocytes	6.8	2.5	%
Eosinophil	6.3	0.2	%
Basophils	0.7	0.2	%
PMN abs	9.93	11.1	1.50 - 7.70 x10^3/uL
Lymphocyte absolute count	2	1.14	1.00 - 4.80 x10^3/uL
Monocyte absolute count	0.96	0.32	0.20 - 1.10 x10^3/uL
Eosinophil absolute count	0.89	<0.10	<=0.50 x10^3/uL
Basophil absolute count	0.1	<0.10	<=0.20 x10^3/uL
Immature granulocyte%	1.9	1.6	0.0 - 1.0 %

**Figure 2:**
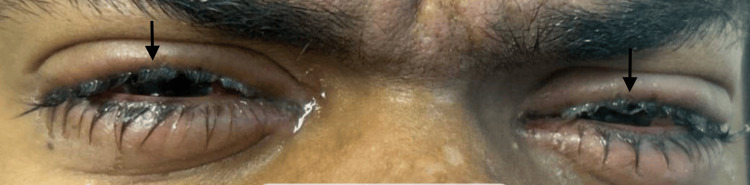
Bilateral conjunctivitis

**Figure 3:**
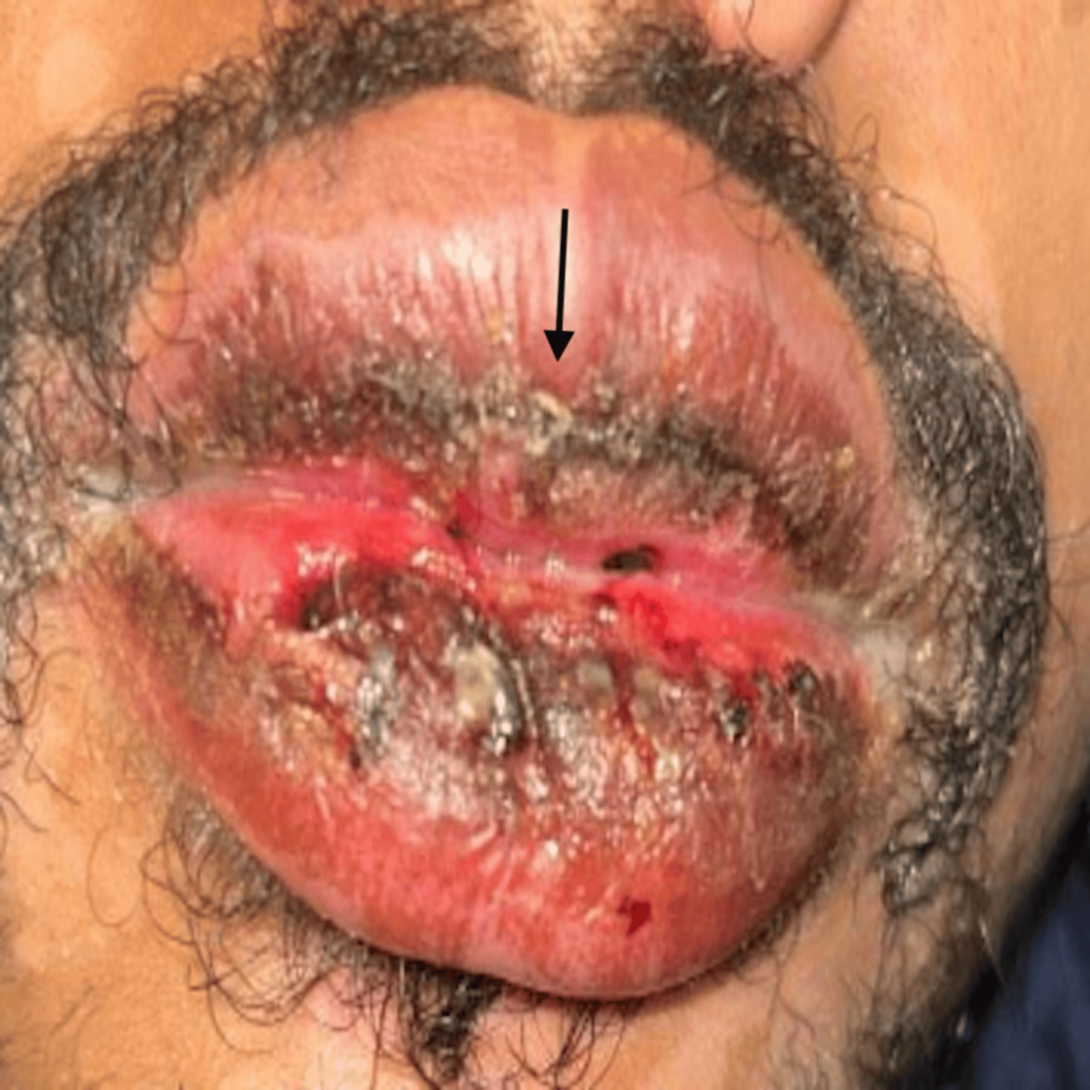
Mucositis

Five days later, the patient was brought back to the emergency department, where he complained of irritation to the eyes, painful mouth sores, and dysuria. He had noticed that the respiratory symptoms had improved somewhat, but the new symptoms were causing increasing discomfort. Therefore, doxycycline was discontinued and azithromycin 500 mg was initiated on Day 1, followed by 250 mg for four days. An EKG conducted prior to starting azithromycin showed normal QTc. On physical examination, it was found that there was bilateral erythema with mild discharge at the conjunctiva (Figure 2), ulcers on the lips that looked shallow (Figure 3), and vesiculobullous lesions present on the hard palate. Additionally, a targetoid rash, nontender and without any pruritus, was observed over both forearms.

Tests at this point revealed an elevated WBC count of 12.8 x 10^9/L, with 86.6% neutrophils and platelets level at 916 x 10^9/L indicating worsening inflammation (Table 1). The urine analysis revealed the presence of ketones at 10 mg/dL and red blood cells at a concentration of 5-10 per high power field (HPF), with no other significant abnormalities detected. A repeat chest X-ray showed near clearing of right upper lobe opacity but with a new development of opacity at the left lung base (Figure 2). He was discharged three days later with a prescription for azithromycin, magic mouthwash including lidocaine for oral mucositis, polymyxin B/trimethoprim eye drops for conjunctivitis, and phenazopyridine for dysuria.

**Figure 4:**
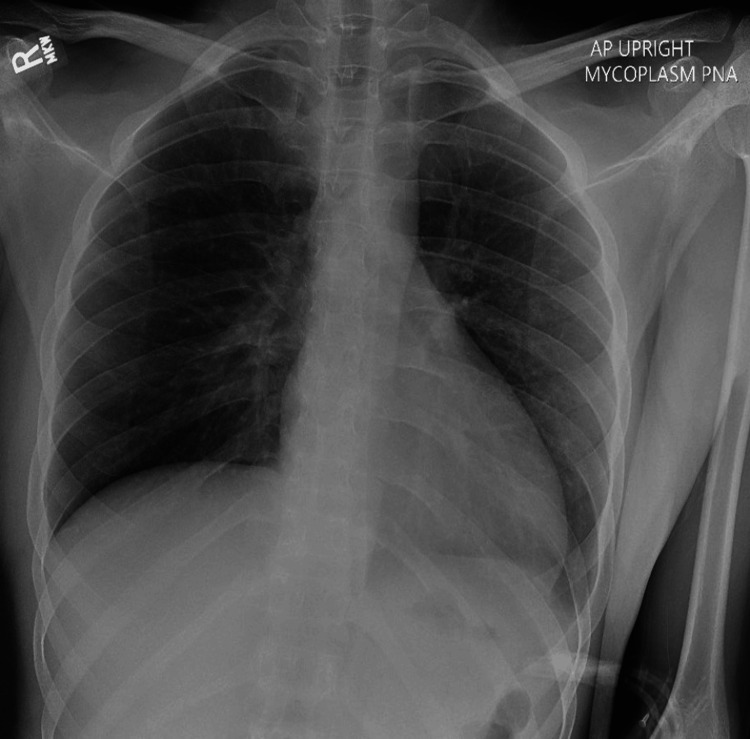
Chest X-ray five days after initial treatment

**Figure 5:**
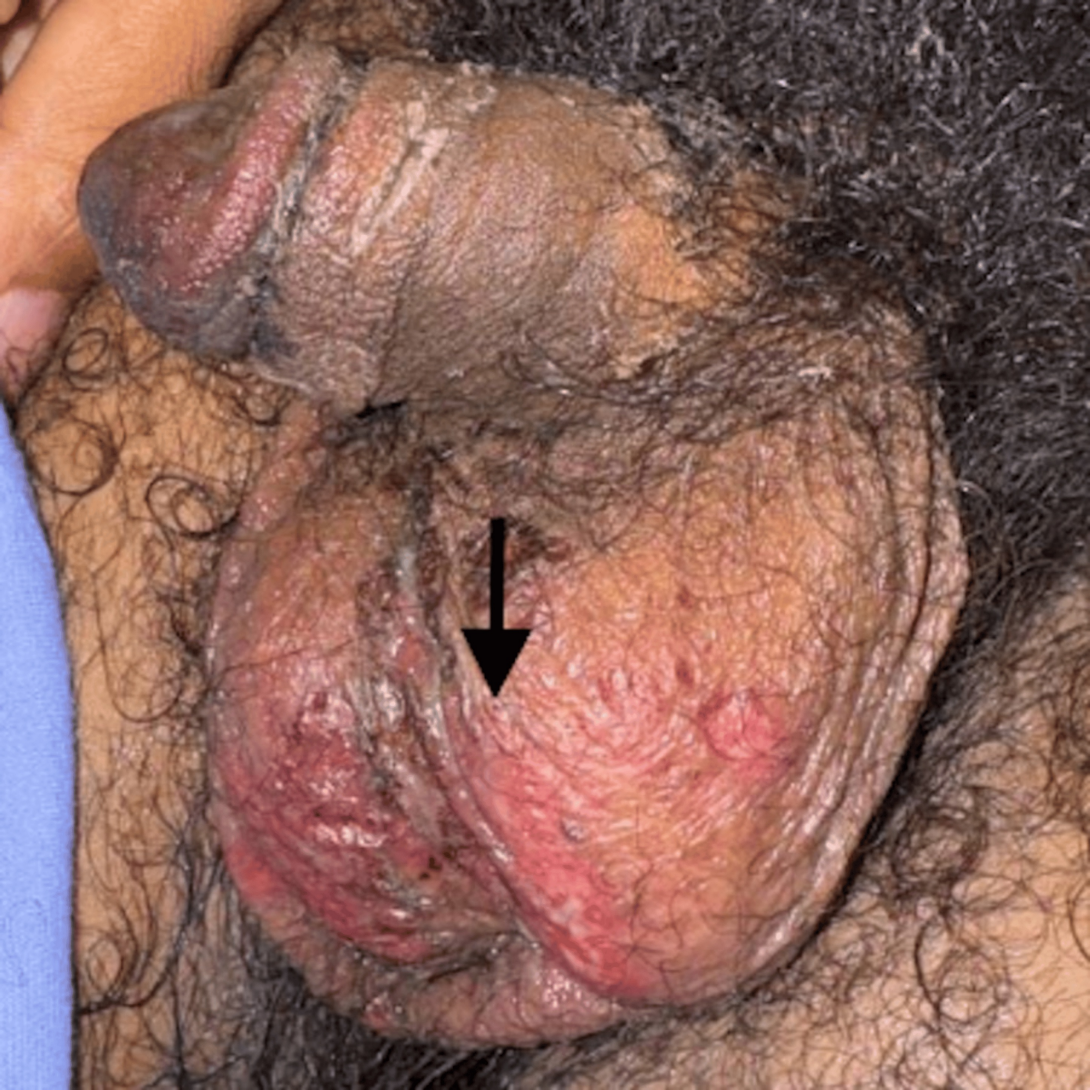
Scrotal erosions

The patient presented three days later with progressive worsening of mucositis with increasing inability to eat and drink and new painful scrotal erosions (Figure 5). Conjunctivitis had persisted with increasing dysuria. Physical examination revealed painful coalescing ulcers of the lips and hard palate and tender erythematous erosions of the scrotum. The targetoid rash on his forearms remained unchanged. Tests showed a sodium level of 133 mg/dL, with creatinine within normal limits. The anion gap was elevated to 14 mmol/L. Urine analysis revealed ketones >150 mg/dL and red blood cells at 10-20 per high power field (HPF). He was admitted for further management due to persistent symptoms despite antibiotic therapy, and 2 liters of normal saline were administered. Given his history and previous diagnosis of *Mycoplasma pneumoniae* infection, a diagnosis of RIME was made.

The patient was started on methylprednisolone 40 mg IV twice per day for severe mucositis and skin involvement. Supportive measures were further continued by using artificial tears for conjunctivitis, topical lidocaine to the scrotal lesions, and hydration. Over three days, he significantly improved with rapid resolution of the conjunctivitis and healing of the oral and scrotal lesions. The patient was discharged with a two-week course of oral prednisone to taper and instructed to follow up with a dermatologist and ophthalmologist for outpatient management.

## Discussion

RIME is a severe, though rare, extrapulmonary complication of *M. pneumoniae*. Due to the generally rare condition and the clinical features somewhat overlapping with most mucocutaneous disorders, it is seldom diagnosed in adults. The clinical manifestations of RIME include mucositis of oral, ocular, and genital mucosa, often associated with targetoid or erythematous skin lesions [6]. These features make it clinically similar to SJS and EM. However, the distinguishing feature is its infectious etiology, usually in bacterial or viral infections such as *M. pneumoniae*, as opposed to drug-related in SJS and EM.

Since RIME is a disorder of immune-mediated pathogenesis, there is speculation that some form of cross-reactive effect between *Mycoplasma* antigens and the tissues in the host might be the most significant trigger of the wide-range mucosal and skin inflammation observed. The patient presented with classic respiratory symptoms, including cough, malaise, and fever, typical of *M. pneumoniae*. The patient's symptoms soon turned out differently when he started to show the most distressing mucocutaneous manifestations, which included painful oral ulcers, conjunctivitis, and scrotal erosions. The non-pruritic targetoid rash on his forearms was less acute but also typical for RIME [7].

However, the crucially important difference between RIME and other mucocutaneous conditions pertains to a critical differentiation in treatment modalities. The primary modes of treatment for SJS and EM include the cessation of the offending drug coupled with supportive care. However, in RIME, antibiotic therapy aimed at the root infection must be combined with systemic anti-inflammatory therapy to rein in the out-of-control immune response [8]. In this case, doxycycline, the first-line antibiotic for *M. pneumoniae*, was administered to the patient. Because the patient's symptoms of respiratory tract illness showed advancement despite antibiotic treatment, he was then switched to azithromycin, commonly used in mycoplasma infections. He continued to worsen from the mucocutaneous symptoms of the disease despite this change. He thus demonstrated that antibiotics were insufficient to control the immune-mediated inflammation underlying RIME.

Corticosteroids like methylprednisolone are one example that has been useful in treating the inflammation component of RIME. This patient indeed responded to the intravenous methylprednisolone with rapid resolution of his conjunctivitis and significant improvement of his oral and scrotal lesions. This aligned with the literature by Xiong et al. (2021), defining corticosteroids as useful adjuvants in managing severe RIME with heavy mucosal involvement [9]. Corticosteroids are immunosuppressive agents that suppress an overactive immune response, which, in turn, permits the healing of skin and mucosal lesions.

Given RIME's disease complexity and seriousness, early recognition and intervention are crucial. Most patients with RIME recover from their disease without sequelae. However, close follow-up is recommended for detection of relapse or development of chronic mucosal inflammation [10]. In this case, the patient was discharged on oral prednisone with a tapering plan and followed up with a dermatologist and ophthalmologist for resolution of all symptoms. Well-treated, this case illustrates how corticosteroid therapy is essential in managing severe RIME and further emphasizes the need for continued research into optimal treatment strategies for this rare condition.

## Conclusions

This case report highlights the effectiveness of early recognition and aggressive corticosteroid therapy in managing RIME in adults. The patient's rapid symptom resolution following steroid treatment demonstrates the importance of prompt diagnosis and appropriate intervention. This case also emphasizes the rarity of RIME in adults, as evidenced by the 24-year-old patient's atypical presentation without respiratory symptoms. The report aims to increase clinician awareness of RIME in adult patients and the importance of involving a multidisciplinary team, potentially improving future diagnosis and treatment outcomes.
